# Right heart flow hemodynamic assessment using 4D flow CMR: a systematic review

**DOI:** 10.1093/ehjimp/qyaf155

**Published:** 2025-12-10

**Authors:** Alexander Gall, Rui Li, Gareth Matthews, Karl-Philipp Rommel, João L Cavalcante, Pankaj Garg

**Affiliations:** Norwich Medical School, University of East Anglia, Norwich, UK; Norwich Medical School, University of East Anglia, Norwich, UK; Norwich Medical School, University of East Anglia, Norwich, UK; Department of Cardiology, Norfolk and Norwich University Teaching Hospitals, Norwich Research Park, Norwich NR4 7UQ, UK; Department of Cardiology, University Medical Centre of the Johannes Gutenberg-University Mainz, Mainz, Germany; German Center for Cardiovascular Research, Partner Site Rhein/Main, Mainz, Germany; Imaging Department, Allina Health Minneapolis Heart Institute, Minneapolis, MN, USA; Norwich Medical School, University of East Anglia, Norwich, UK; Department of Cardiology, Norfolk and Norwich University Teaching Hospitals, Norwich Research Park, Norwich NR4 7UQ, UK

**Keywords:** right atrium, right ventricle, cardiovascular magnetic resonance imaging

## Abstract

Right heart dysfunction increases morbidity and mortality in cardiovascular diseases. Four-dimensional flow cardiovascular magnetic resonance (4D flow CMR) imaging evaluates detailed right heart physiology, including vorticity, flow dynamics, kinetic energy (KE) and energy loss (EL). This systematic review synthesized literature using 4D flow CMR to assess right atrial (RA) and right ventricular (RV) hemodynamics in health and disease. A systematic search of the Scopus database (up to March 2025) identified observational studies investigating 4D flow CMR of right heart function in adults. Data on RA flow dynamics, RV flow components, KE, EL, and hemodynamic parameters were narratively synthesized. Quality assessment used the AXIS tool From 240 identified articles, 17 studies (894 participants) met eligibility criteria, including healthy individuals and patients with pulmonary hypertension (PH). RA flow dynamics, described in five studies, were characterized by a dominant vortex in health, interrupted with disease. RV flow components consistently showed a decline in direct flow and increased residual volume with disease. Atrial and ventricular KE assessments revealed age, sex, and disease-specific alterations, with rotational flow appearing to conserve right atrial KE. Increased EL was noted in PH. 4D flow CMR is a powerful tool for assessing novel right heart hemodynamic parameters. Quantifying flow patterns, components, and energetics provides a comprehensive overview of right heart function, promising to improve the diagnosis, management, and prognostic stratification of right heart diseases.

## Introduction

Interest in right heart function in health and disease has experienced a resurgence in recent years, driven by the increasing recognition that right heart dysfunction is intrinsically linked to increased morbidity and mortality across a spectrum of cardiovascular conditions.^1^ This marks a significant shift from the historical perspective, which often underestimated the right heart’s importance, sometimes viewing it as a passive conduit or even dispensable.^2–4^ The right heart, comprising the right atrium (RA) and right ventricle (RV), functions as an integral part of the low-pressure, high-compliance cardiopulmonary circuit.^5–7^ The right atrium acts as a crucial reservoir and conduit for systemic venous return, primarily receiving deoxygenated blood via the superior and inferior vena cavae, as well as the coronary sinus (CS).^8,9^ During ventricular diastole, this blood traverses the tricuspid valve to fill the right ventricle, which subsequently exits through the right ventricular outflow tract into the pulmonary arterial circulation.^7,10,11^

Physiologically distinct from the left ventricle (LV), the RV operates with ∼20% of the LV mass and expends only about one-sixth of the energy, yet maintains an equivalent cardiac output under normal conditions.^1,12^ This efficiency underscores the critical role of flow dynamics and the preservation of kinetic energy in a normally functioning right heart.

Chronic RV dysfunction most commonly develops in response to increased afterload; frequently stemming from pulmonary hypertension (PH), which itself is often secondary to left heart failure.^1^ However, the RV, much like the LV, is susceptible to failure under conditions of sustained pressure or volume overload; significant volume overload, for instance, often results from severe tricuspid valvular incompetence.^13^ The RV initially adapts to such stressors through compensatory remodelling mechanisms like hypertrophy and hypercontractility to maintain adequate cardiac output.^14^ Yet, prolonged hemodynamic stress leads to negative remodelling, typically characterized by diastolic dysfunction, chamber dilation, wall thinning, and fibrosis, ultimately culminating in contractile dysfunction and RV to pulmonary artery uncoupling.^13,15^

Preservation of right heart flow is therefore important to maintain the low resistance, high compliance system of the cardiopulmonary venous unit.^1^ The efficiency of this system relies on the combined effects of sustained forward flow of systemic venous return, maintenance of laminar blood flow through the right heart, and low outflow resistance, as evidenced by minimal RV afterload.^7^ Together, these factors preserve kinetic energy, minimize energy loss, and support adequate RV function.

Assessing *in vivo* these complex, three-dimensional flow dynamics non-invasively has become feasible with four-dimensional (4D) flow cardiac magnetic resonance (CMR) imaging, which captures blood velocity in three spatial dimensions resolved over time.^16,17^ While early applications of flow CMR, including 4D flow, focused on improving assessments of valvular function and shunt quantification compared with traditional methods like transthoracic echocardiography (TTE), recent research has increasingly shifted towards deriving and analysing novel hemodynamic parameters.^16,17^ These include detailed quantification of intracardiac flow component pathways, kinetic energy fluctuations throughout the cardiac cycle, vorticity, hemodynamic forces, and energy loss estimations.^16–19^ Studies utilizing 4D flow CMR have begun to quantify right heart flow hemodynamics, examining flow patterns in both healthy and diseased states to determine their value in assessing kinetic energy, energy loss and maladaptive changes associated with disease.^9,16^

This review aims to systematically synthesize the existing literature that utilizes 4D flow CMR for the assessment of right heart flow hemodynamics. We will explore the reported findings on flow patterns, flow components, kinetic energy, energy loss, and other relevant hemodynamic parameters within the right atrium and ventricle, comparing observations in healthy populations with those in various pathological conditions.

## Methods

This systematic review was prospectively registered with the International Prospective Register of Systematic Reviews (PROSPERO) (CRD420251028567). The Preferred Reporting Items for Systematic Reviews and Meta-Analysis (PRISMA) guidelines were followed for study selection, review process and evidence synthesis.^20^

### Eligibility criteria

Studies that investigated the role of 4D flow CMR in the assessment of right atrial or right ventricular hemodynamic function were eligible. English language, human research studies were included if subjects were >18 years of age. Studies with complex congenital heart disease were excluded, as were case studies, methodological studies and review articles. Those without specific research outcomes or not focusing on an aspect of right heart hemodynamics were also excluded.

### Search strategy and study selection

A comprehensive search was conducted using the Scopus database, which includes majority of studies indexed on MEDLINE and also whole of EMBASE, on 17 March 2025.^21^ The search was limited to articles published in English and included the following search terms: (TITLE-ABS-KEY (‘right heart flow’) OR TITLE-ABS-KEY (‘right atrial flow’) OR TITLE-ABS-KEY (‘right ventricular flow’) OR TITLE-ABS-KEY [‘4D FLOW’ AND (‘right atrial’ OR ‘right atrium’ OR ‘right ventricle’ OR ‘right ventricular’)] OR TITLE-ABS-KEY (‘4D FLOW’ AND ‘right heart’)).

**Figure 1:**
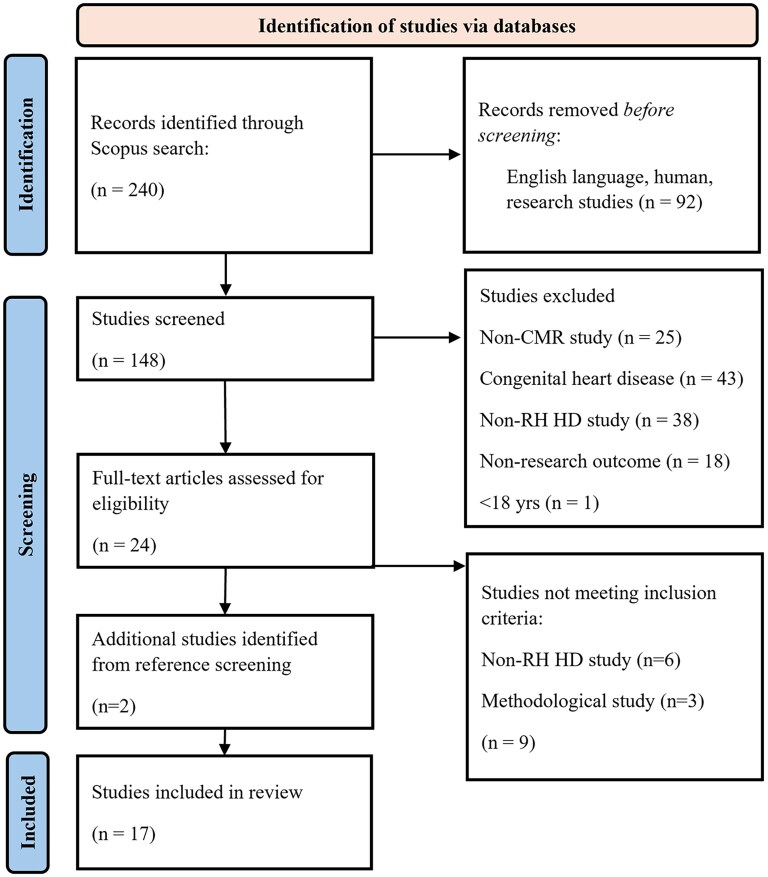
PRISMA flow chart of literature search.^20^ Non-RH HD study, non-right heart hemodynamic study.

The selection process was performed in two stages. Initially, one author (AG) screened titles and abstracts of retrieved studies to assess eligibility. Any studies that did not clearly meet the inclusion criteria were discussed with a second author (PG). In the second stage, full-text articles were reviewed against inclusion criteria by one author (AG) and discussed with a second author (PG). References of all included articles were screened for further studies that may meet inclusion criteria (*Figure 1*). Data extraction and risk of bias analysis were performed by one author (AG).

### Quality assessment

Risk of bias was assessed using the Appraisal tool for Cross-Sectional Studies (AXIS tool).^22^ The AXIS tool consists of 20 components, designed to assess bias and aid the inclusion of cross-sectional studies in systematic reviews. The AXIS tool was chosen as most studies are cross-sectional in design.

### Data synthesis

Given the anticipated heterogeneity of the populations studied, CMR methodologies and parameters reported, the primary data synthesis is a narrative summary of results. Results are synthesized as coherent and consistent themes reported across the included studies, relevant to the hemodynamics of the right heart. Where appropriate quantitative data is included in the review.

## Results

### Search results

**Figure 2:**
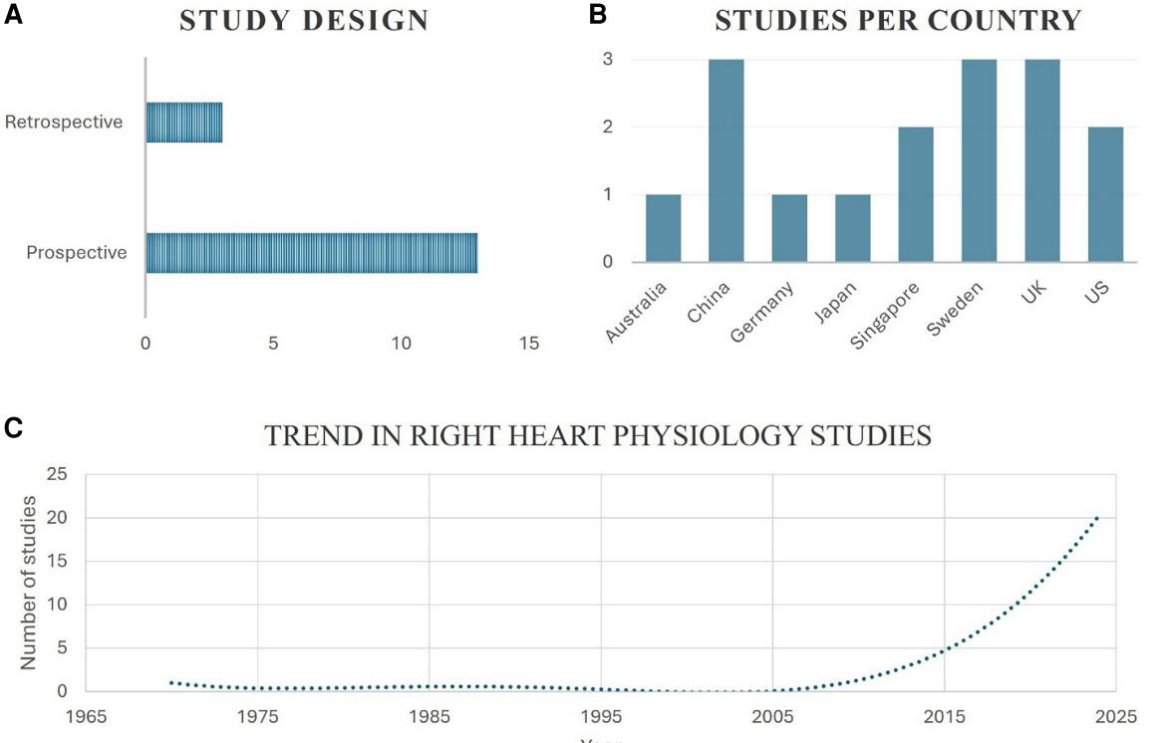
Overview of study characteristics. This includes (*A*) the design of the studies included in this systematic review, (*B*) the country of origin of these studies, and (*C*) the trend in the number of studies investigating right heart physiology over time, based on the 148 studies screened for eligibility.

This systematic review identified 17 studies investigating right heart hemodynamics using 4D flow CMR. Across these 17 studies, a total of 894 participants were included, encompassing healthy volunteers and patient populations relevant to right heart assessment [predominantly pulmonary hypertension, including chronic thromboembolic pulmonary hypertension (CTEPH) and pulmonary arterial hypertension (PAH)]. All patients were in sinus rhythm. *Table 1* provides a summary of the included studies, while *Figure 2* details study characteristics. The following sections synthesize the findings from these studies concerning right atrial flow dynamics, right ventricular flow components, kinetic energy, and energy loss.

**Table 1:** Summary table of the studies included in this systematic review

First author (year)	Country	Study design	Cohort	*n*	Female (%)	Mean ± SD age (years)	Outcome focus
Arvidsson (2013)^23^	Sweden	Prospective	Healthy adults	15	46.7	23–52^b^	RA and LA kinetic energy
Arvidsson (2017)^5^	Sweden	Prospective	Healthy adults	25	53.8	28 (23–63)^a^	Hemodynamic forces
			Elite athletes	14		24 (18–30)^a^	
			LBBB	2		58 & 72	
Barker (2020)^19^	UK Netherlands	Prospective	Healthy adults	53	39.6	45 ± 17	RV kinetic energy
Callaghan (2017)^8^	Australia	Prospective	Healthy adults	12	33.3	40 ± 13	RA flow dynamics Kinetic energy
Dewhurst (2020)^9^	UK	Prospective	Healthy adults	18	61.1	21 to 50^b^	RA flow patterns Kinetic energy Energy loss
Fenster (2015)^24^	USA	Prospective	PAH	13	73.9	63 ± 8	RV/RA flow dynamics
			Healthy controls	10		58 ± 10	
Fredriksson (2011)^10^	Sweden	Prospective	Healthy adults	10	40.0	46 ± 17	RV flow components Kinetic energy
Fredriksson (2016)^25^	USA	Prospective	IHD/lower LV remodelling	11	57.6	71 ± 4	RV flow components Kinetic energy
			IHD/higher LV remodelling	11		69 ± 5	
			Healthy controls	11		67 ± 4	
Han (2015)^6^	Sweden	Prospective	PAH	10	73.7	48 ± 14	RV kinetic energy PA viscous energy loss
			Healthy controls	9		30 ± 13	
Nakaji (2021)^11^	Japan	Prospective	Healthy adults	19	42.1	30 ± 5	RV flow dynamics Energy loss Kinetic energy
Parikh (2017)^26^	UK	Prospective	Healthy adults	13	53.8	41 (25–50)^a^	RA flow dynamics
			Cryptogenic Stroke-PFO	13		40 (21–50)^a^	
Wang (2021)^27^	China	Retrospective	PAH	30	56.8	49 ± 13	RV flow components
			Healthy controls	14		44 ± 12	
Wehrum (2018)^28^	Germany	Prospective	Age-related population-based sample	126	50.8	Age-group 20–39: 30 ± 5 Age-group 40–59: 50 ± 6 Age-group 60–80: 69 ± 5	RA flow dynamics
Xu (2022)^29^	China	Retrospective	CTEPH	67	29.9	48 ± 14	RV flow components
Xu (2023)^30^	China	Retrospective	Pre-PH	105	51.2	49 ± 13	Biventricular flow components
			Healthy controls	24		40 ± 12	
Zhao (2022)^31^	Singapore	Prospective	PAH	45	75.0	46 ± 11	RV flow components Kinetic energy
			Healthy controls	51		46 ± 14	
Zhao (2023)^32^	Singapore	Prospective	Healthy adults	163	41.7	43 ± 13	Biventricular flow components Kinetic energy

^a^Median (IQR).

^b^Range.

Cryptogenic Stroke-PFO, cryptogenic stroke with patent foramen ovale; CTEPH, chronic thromboembolic pulmonary hypertension; IHD, ischaemic heart disease; LA, left atrium; LBBB, left bundle-branch block; LVEDVI, indexed left ventricular end diastolic volume; PA, pulmonary artery; PAH, pulmonary arterial hypertension; Pre-PH, pre-capillary pulmonary hypertension; RA, right atrium; RV, right ventricle; SD, standard deviation.

### Right atrial flow dynamics

**Figure 3:**
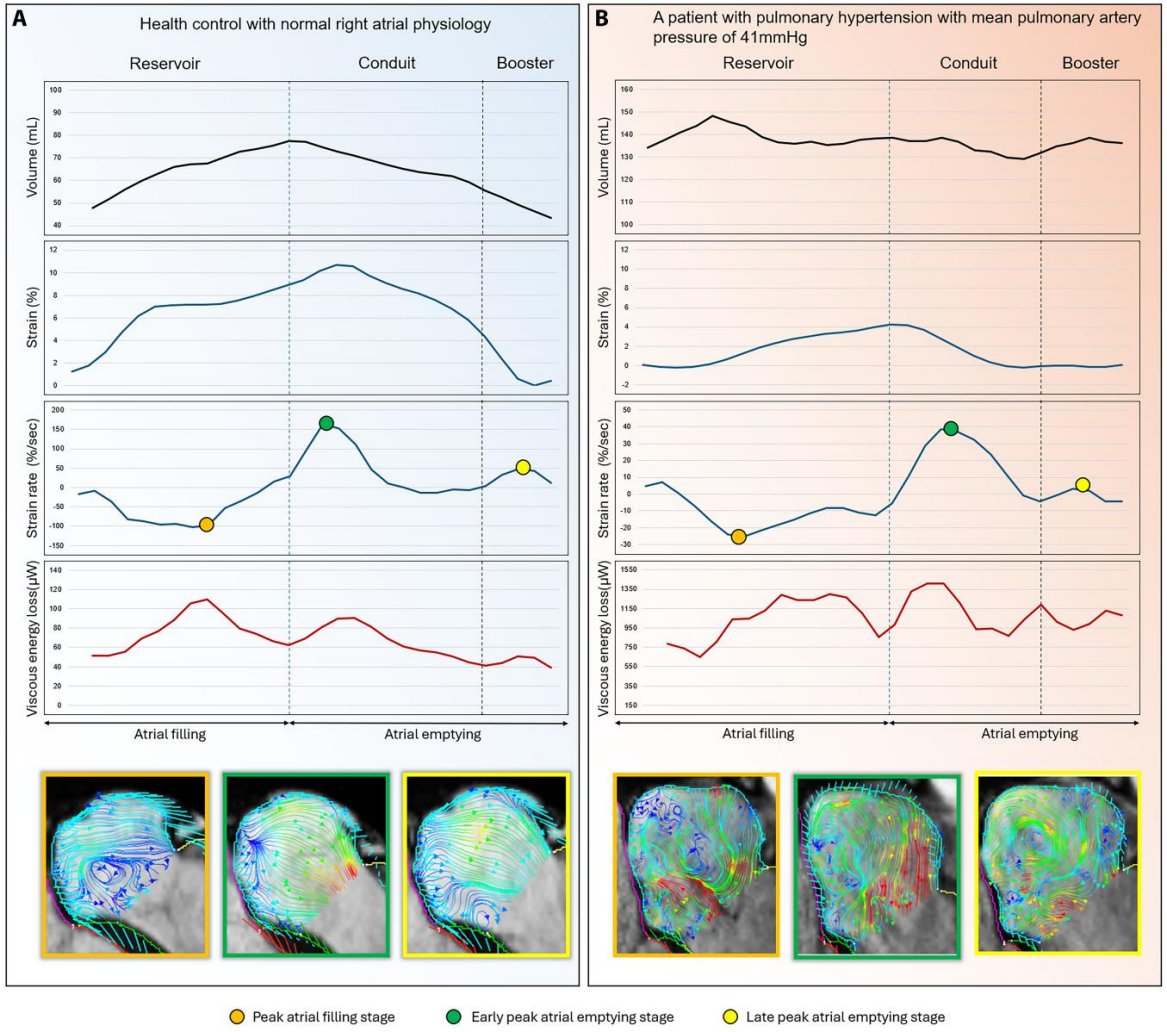
Phasic right-atrial mechanics and intracavitary flow in health and pulmonary hypertension. Time-resolved right-atrial (RA) volume, longitudinal strain, strain rate (SR), and 4D-flow–derived viscous energy loss (VEL) are shown across the reservoir, conduit, and booster phases (vertical dashed lines). (*Panel A*): healthy control. (*Panel B*): pulmonary hypertension (PH) with mean pulmonary artery pressure (mPAP) 41 mmHg. Coloured markers denote sampling instants—peak atrial filling (yellow), early peak atrial emptying (green), and late peak atrial emptying (yellow) with phase-matched 4D-flow streamline maps displayed below each graph. Axes: volume (mL), strain (%), SR (%·s⁻¹), and VEL (µW). RA, right atrium; PH, pulmonary hypertension; mPAP, mean pulmonary artery pressure; SR, strain rate; CMR, cardiovascular magnetic resonance; 4D flow, time-resolved three-dimensional phase-contrast CMR; VEL, viscous energy loss.

Five studies utilized 4D flow CMR to characterize right atrial flow dynamics.^8,9,24,26,28^ This was carried out in both healthy individuals and specific patient populations. In healthy adults, RA flow was often dominated by a single, large, clockwise rotating vortex, which was primarily driven by IVC inflow, with complementary SVC inflow facilitating vortex formation through avoidance colliding streams (*Figure 3*).^8,9^ Callaghan *et al.*^8^ identified that 79% of total RA blood flow is made up of the primary vortex which acts to preserve kinetic energy. Furthermore, the majority of RA inflow (74%) passes through the RA within a single cardiac cycle.^8^ Vortical flow predominantly entered the right atrium early, while non-vortical flow entered late and had a low residence time while traversing the RA.^8^ Dewhurst *et al.*^9^ confirmed this single organized vortex in most (13/18) of their healthy cohort, with four showing multiple vortices and one not demonstrating any organized vortex formation, linking lower IVC helicity or IVC kinetic energy (KE) flux dominance to formation of a single vortex.^9^

As with Dewhurst *et al.*,^9^ Parikh *et al.*^26^ highlighted the heterogeneity of RA flow patterns including vortical, helical, helico-vortical and multiple vortices. Parikh *et al.*,^26^ comparing healthy controls with patients having a cryptogenic stroke in the presence of a patent foramen ovale (PFO), found that a single organized vortex was significantly less common in the cryptogenic stroke-PFO group (2 vs. 8 controls).^26^ They correlated this finding with anatomical variations, noting a significantly greater right-left separation between the SVC and IVC entry points in the cryptogenic stroke-PFO group, suggesting that caval arrangement significantly impacts RA flow organization which could, in the long term, associate with RA remodelling.^26^ Wehrum *et al.*^28^ demonstrated age-related alterations in RA flow dynamics in their population-based study (*n* = 126).^28^ The majority of individuals <60 years of age demonstrated a single organized vortex (79/86), whereas in those ≥60 years, there was significantly more flow turbulence, with 12/39 subjects demonstrating no rotational RA blood flow.^28^ The authors suggested this may relate to observed age-related anatomical changes, including lateralization of the caval vein axes and increased RA volumes.^28^

The potential impact of CS flow on overall right atrial flow dynamics was considered minimal in one study.^9^ This conclusion was based on the substantially lower CS flow rate in health (1.5 ± 0.6 mL s^−1^) compared with that of the SVC (40.3 ± 14.1 mL s^−1^) and IVC (62.5 ± 17.3 mL s^−1^). Due to its anatomical proximity to the IVC opening, the study hypothesized that CS blood flow is likely entrained by the much stronger IVC stream and was therefore not investigated further.^9,33^

Fenster *et al.*^24^ performed a quantitative vorticity analysis of both RA and RV vorticity as a marker of RV diastolic dysfunction (RVDD), in both PAH patients and healthy controls, comparing CMR-derived markers of RVDD with TTE. They demonstrated significantly increased A-wave vorticity and decreased E-wave vorticity in RVDD compared with controls, with these markers correlating to TTE markers of RVDD.^24^

### Right ventricular flow components

The RV blood volume was categorized into four flow components: direct flow (DF), retained inflow (RI), delayed ejection flow (DEF) and residual volume (RVo).^10^ Early research by Fredriksson *et al.*^10^ in 2011 used 4D flow CMR pathline visualization to map intracardiac blood flow, describing the RV DF component as a smooth curve through the basal and mid-ventricular portions, largely excluding the apex.^10^ The RV DF had a larger volume than other flow components and exhibited a larger volume as a percentage of end-diastolic volume (EDV) than the LV DF. However, RV RVo represented a smaller volume when expressed as a percentage of EDV than LV RVo.^10^ Zhao *et al.*^32^ reported flow components in a cohort of 163 healthy individuals, reporting median values as 35%, 17%, 16%, and 30% of EDV, respectively for DF, RI, DEF and RVo.^32^ They also report values for the LV of 34%, 15%, 17% and 33% respectively, which are much closer than those reported by Fredriksson *et al*.^10,32^ Zhao *et al.*^32^ also found significant sex differences in RV flow components, with women exhibiting lower median RV RVo (27% vs. 33%) and higher median RV DF (38% vs. 34%). There was no significant impact of ageing on RV flow components found in this cohort.^32^

In patients with mild ischaemic heart disease and LV remodelling, Fredriksson *et al.*^25^ demonstrated a decrease in RV DF with higher levels of LV remodelling when compared with early remodelling and healthy controls, while there were no significant differences on TTE or CMR with conventional markers of RV dysfunction.^25^ Similarly, in three studies focussing on patients with pulmonary hypertension, RV DF was significantly decreased, while RV RVo was significantly increased when compared with controls.^27,30,31^ Xu *et al.*^30^ not only demonstrated that RV DF and RVo correlated with markers of RV function and remodelling, but also demonstrated good sensitivity and specificity for predicting mean pulmonary artery pressure (mPAP) ≥ 25 mmHg with thresholds of RV DF <11% and RVo >42%.^30^ Wang *et al.* and Zhao *et al.*^31^ similarly demonstrated a correlation of RV flow components with markers of RV dysfunction, remodelling and pulmonary vascular resistance (PVR) in cohorts of patients with PAH, finding a decrease in RV DF and an increase with RV RVo compared with healthy controls.^27,31^ Zhao *et al.*^31^ further demonstrated a correlation between RV DF and RVo, and exercise capacity as measured by cardiopulmonary exercise testing in patients with PAH.^31^ Finally, Xu *et al.*^29^ demonstrated median values of RV DF, DEF, RI and RVo of 18%, 15%, 16% and 51% respectively in a cohort of 67 patients with CTEPH.^29^  *Table 2* further summarizes the reported parameters of RV flow components.

**Table 2:** Right ventricular flow components, as reported by seven studies

First author (year)	Cohort	n	DF (%)	RI (%)	DEF (%)	RVo (%)
Fredriksson (2011)^10^	Healthy adults	10	44 ± 6	17 ± 3	15 ± 3	23 ± 6
Fredriksson (2016)^25^	Healthy controls	11	44 ± 6	19 ± 3	13 ± 3	24 ± 7
	IHD/lower LV remodeling	11	44 ± 6	17 ± 4	17 ± 5	21 ± 4
	IHD/higher LV remodeling	11	38 ± 5	21 ± 5	18 ± 3	24 ± 5
Wang (2021)^27^	Healthy controls	14	41 ± 3	19 ± 2	20 ± 2	20 ± 5
	PAH	30	16 ± 9	17 ± 6	16 ± 6	51 ± 16
Xu (2022)^29^	CTEPH	67	18 (13)^a^	16 (7)^a^	15 (3)^a^	51 (24)^a^
Zhao (2022)^31^	Healthy controls	51	37 (7)^a^	16 (6)^a^	17 (5)^a^	29 (10)^a^
	PAH	45	24 (16)^a^	16 (5)^a^	14 (6)^a^	44 (16)^a^
Xu (2023)^30^	Healthy controls	24	31 ± 11	23 ± 4	22 ± 3	23 ± 10
	Pre-PH	105	6 ± 9	16 ± 6	15 ± 6	63 ± 18
Zhao (2023)^32^	Healthy adults	163	35 (10)^a^	17 (6)^a^	16 (5)^a^	31 (11)^a^
	Healthy males	95	34 (8)^a^	17 (5)^a^	15 (6)^a^	33 (9)^a^
	Healthy females	68	38 (10)^a^	17 (6)^a^	17 (7)^a^	27 (11)^a^

Reported as mean ± standard deviation unless denoted otherwise. Flow components are expressed as a percentage of right ventricular end-diastolic volume.

^a^Median (interquartile range).

CTEPH, chronic thromboembolic pulmonary hypertension; DEF, delayed ejection flow; DF, direct flow; IHD, ischaemic heart disease; LV, left ventricle; PAH, pulmonary arterial hypertension; Pre-PH, pre-capillary pulmonary hypertension; RI, retained inflow; RVo, residual volume.

### Kinetic energy

KE of intracardiac blood flow was quantified across nine studies, indexed to EDV (KEiEDV).^6,8–11,19,23,25,31^ In healthy subjects, Fredriksson *et al.*^10^ found that the RV DF component possessed significantly higher KE than the other RV flow components at end-diastole.^10^ Zhao *et al.*^32^ and Barker *et al.*^19^ reported reference values for RV KEiEDV, both reporting changes in diastolic KE with ageing.^19,32^ Both studies demonstrated a decline in peak E-wave KEiEDV and an increase in peak A-wave KEiEDV with age, with a subsequent significant decrease in the KEiEDV E/A ratio. However, the reported mean values differed between the two papers [KEiEDV (Barker vs. Zhao) peak E-wave (5.53 vs. 13.4 μJ/mL), peak A-wave (4.59 vs. 9.2 μJ/mL) and KEiEDV E/A ratio (1.51 vs. 1.80)], which the authors postulated was due to sample size and differences in RVEDV size in Asian vs. Caucasian ethnicities.^19,32^ Zhao *et al.*^32^ also reported sex differences, with women exhibiting higher median RV global, RV average diastolic and RV peak E-wave KEiEDV.^32^

Alterations in KE were seen in disease states, with Han *et al.* reporting higher KE work density and Zhao *et al.*^31^ reporting significantly decreased peak E-wave KEiEDV in PAH patients compared with healthy controls.^6,32^ Fredriksson *et al.*^25^ found that the KE possessed by the RV DF component relative to the total EDV KE (DF/EDV KE-ratio) was significantly lower in patients with primary LV disease and greater LV remodelling.^25^

Characterizing atrial energetics in healthy individuals, Arvidsson *et al.*^23^ used 4D flow CMR in 15 healthy volunteers to specifically quantify RA and LA KE throughout the cardiac cycle.^23^ They found that mean RA KE was significantly higher than mean LA KE (1.7 ± 0.1 mJ vs. 1.1 ± 0.1 mJ, *P* < 0.01). Three KE peaks were observed in both atria: during ventricular systole, early ventricular diastole (E-wave), and atrial contraction (A-wave).^23^ Notably, the systolic RA KE peak was significantly larger than the systolic LA KE peak, while the early diastolic LA KE peak was larger than the RA equivalent. Rotational flow conserved energy better than non-rotational flow. In the LA, early diastolic KE correlated with LV mass, while in the RA there was no correlation between RV relaxation and atrial KE.^23^

### Energy loss and other hemodynamic parameters

Nakaji *et al.*^11^ assessed energy loss (EL) within the right heart in healthy individuals, using 4D flow CMR. They reported EL peaks during systole (in the right ventricular outflow tract/PA) and diastole (within RV vortices).^11^ The mean EL index for the right heart was 0.63 ± 0.16 mW/m², lower than that reported for the LV system (1.02 ± 0.26 mW/m²).^11^ Han *et al.* measured viscous energy loss specifically in the main PA, finding it was significantly elevated in PAH patients compared with controls (21.1 ± 6.4% vs. 2.2 ± 1.3% of KE output, respectively).^6^

Finally, Arvidsson *et al.*^5^ computed 3D hemodynamic forces in healthy subjects and elite athletes using 4D flow CMR. RV forces were found to be both longitudinal and axial, in contrast to the predominantly longitudinal forces seen in the LV. This caused a ‘slingshot-like acceleration’ when transitioning blood from RV inflow to outflow.^5^

## Discussion

**Figure 4:**
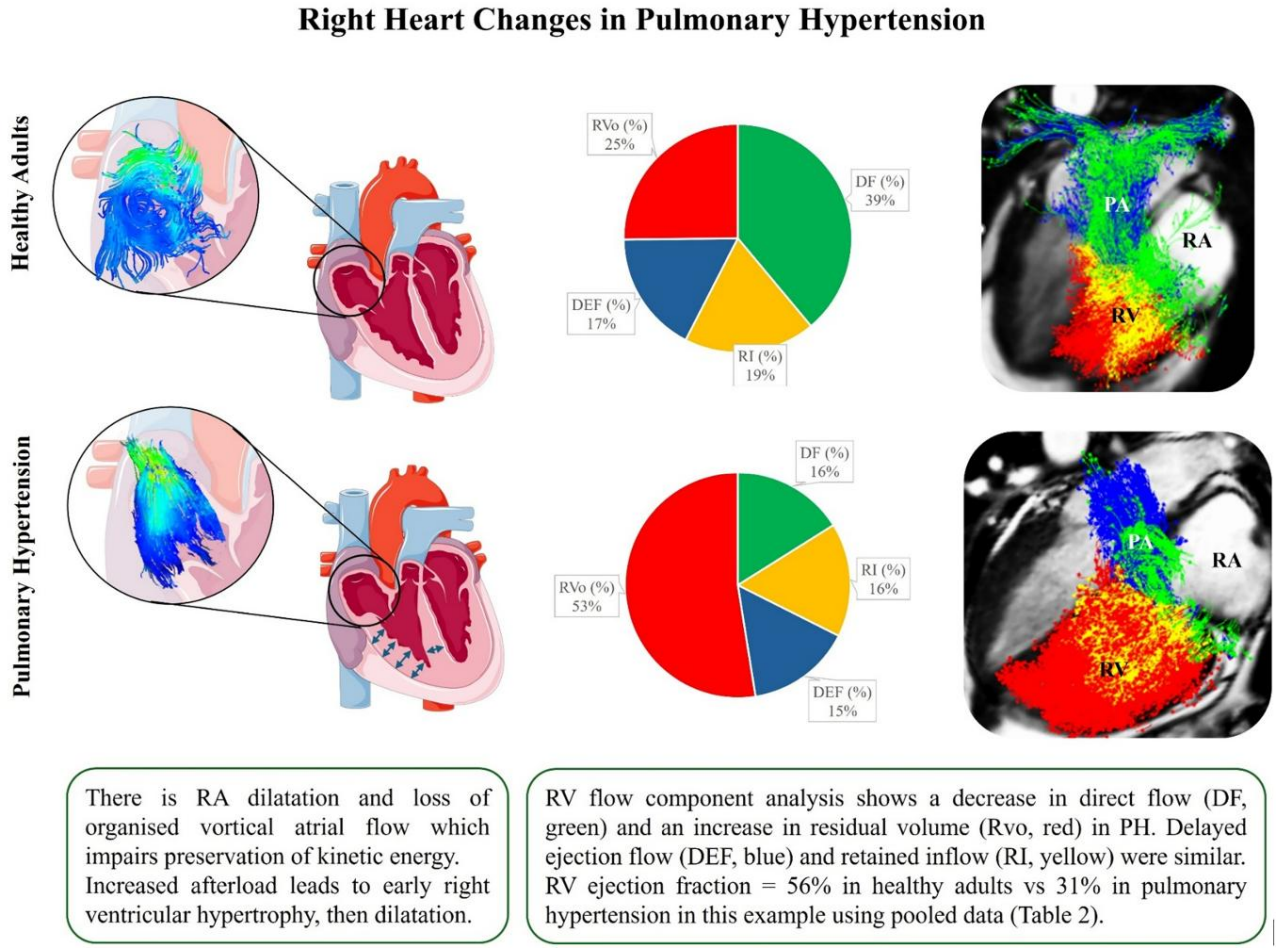
Right heart flow changes in pulmonary hypertension (PH). The Pie Chart shows the components of right ventricular flow and how residual volume increases significantly in PH, thereby affecting right ventricular hemodynamics. RA, right atrium; RV, right ventricle; PA, pulmonary artery.

This systematic review synthesized findings from 17 studies utilizing 4D flow CMR to assess right heart hemodynamics. While the total number of patients encompassed 894 individuals (including healthy volunteers and patients primarily with pulmonary hypertension subtypes such as PAH and CTEPH), most studies had a small sample size, using different methodologies for post-processing which creates heterogeneity in the assessment of the parameters measured. Nonetheless, it demonstrates the capability of 4D flow CMR to visualize and quantify complex flow patterns and derive novel hemodynamic metrics within the RA and RV (*Figure 4*). Key findings across these studies reveal characteristic flow patterns in health, including a dominant clockwise RA vortex and distinct RV flow components, which become significantly altered in disease states, particularly pulmonary hypertension. Specifically, reduced RV DF and increased RV RVo emerged as consistent features in PH, correlating strongly with conventional markers of RV dysfunction, adverse remodelling, and invasive hemodynamic parameters (e.g. mPAP, PVR). Kinetic energy analyses highlighted age- and sex-related differences and disease-specific alterations, particularly in diastolic KE parameters. Furthermore, energy loss parameters were quantified, showing deviations from normal in disease states and therefore highlighting potential clinical utility.

### Right atrial dynamics

The RA exhibits complex and functionally significant hemodynamics as illustrated by 4D flow CMR. The frequent observation of a single, dominant, rotating vortex in healthy individuals appears central to efficient atrial function, resulting from the interaction of IVC and SVC inflow to maintain organized flow (*Figure 3*). As suggested by Callaghan *et al.*,^8^ this vortex, involving the majority of RA throughput, likely facilitates the smooth redirection of caval inflow towards the tricuspid valve while preserving KE, potentially minimizing the work required for RV filling.^8^

However, this review highlights the heterogeneity of RA flow patterns, even in health, which challenges the idea of a single organized vortex as the only ‘normal’ pattern. The work by Dewhurst *et al.*^9^ and Parikh *et al.*^26^ links this heterogeneity (including helical, helico-vortical, and multiple vortex patterns) to anatomical variations, particularly the relative positioning and inflow characteristics of the caval veins.^9,26^ It is not clear, however, whether these anatomical and functional variations confer any long-term risk with regard to RA remodelling and RV function. However, early surgical trials demonstrated improved energy loss characteristics with *in vitro* experiments replicating caval offset when compared with aligned caval inflow, describing an organized vortex most prominent at an offset of 0.5 the diameter of the caval veins, which was lost with increasing caval separation.^34^

Parikh *et al.*^26^’s findings further raise questions about whether increased caval separation predisposes to non-vortical flow patterns while also impacting on right to left shunting in those with a PFO and therefore increases risk of paradoxical embolism.^26^ Beyond the influence of the caval veins and PFOs, the impact of the CS on RA flow dynamics also warrants consideration. CS flow and coronary flow reserve can be altered in a number of disease states, affecting RA venous return and pressure.^35^ The clinical relevance of this is amplified by the increasing use of interventions that directly modulate CS flow, including the CS reducer and left atrial-to-CS shunt device.^36,37^

The age-related decline in organized vortical flow and increase in turbulence reported by Wehrum *et al.*^28^ represents a key finding. In keeping with the findings from Parikh *et al.*,^26^ this may be predisposed by changes in caval vein arrangement, this time due to age, raised filling pressures and subsequent RA remodelling rather than anatomical variation.^26,28^ However, it is also possible that the RA remodelling could be a consequence of changes in flow dynamics rather than the causative factor. The work of Fenster *et al.*^24^ linked markers of RVDD with alterations in RA flow dynamics, including decreased E-wave and increased A-wave vorticity, further linking alterations in RA flow dynamics with right heart function.^24^ Better understanding of RA flow dynamics is crucial for interpreting right heart function. As organized vortical flow is a key mechanism for kinetic energy preservation, the chaotic activation of atrial fibrillation (AF) likely disrupts this vortex, resulting in significant energy dissipation. Moreover, if altered flow patterns driven by caval vein anatomy predispose to RA remodelling, the chronic hemodynamic disturbance of AF represents a plausible parallel mechanism driving the structural maladaptation observed in these patients.

### Right ventricular hemodynamics

The categorization of RV function into four flow components provides a consistent framework for understanding the efficiency of the RV. Furthermore, it is conceptually applicable to currently used markers of RV systolic function, with DF plus DEF correlating with RVEF, whilst also providing more granular detail.^27,29^ The consistently reported metrics in disease indicated a reduction in effective ventricular throughput with a rise in the static component (decreased DF/increased RVo), representing a failing ventricle. These markers were strongly correlated with more established metrics including right ventricular ejection fraction, RV volumes, and RV global longitudinal strain.^27,29,31^

To add strength to the utility of the RV flow components, they were also well correlated to invasive hemodynamic measures including PVR and mPAP. This includes the preliminary work by Xu *et al.*^29^ indicating potential thresholds of DF and RVo to predict raised pulmonary pressures, further strengthening the clinical utility of these parameters.^29^

### Energetics

The addition of energetics assessments with KE and EL adds a further dimension to 4D flow CMR for a comprehensive understanding of right heart function. The changes in KE described by Zhao *et al.*^32^ and Barker *et al.*^19^ interestingly mirror the findings from Fenster *et al.*^24^ regarding RA vorticity, implying that the KE of the blood pool within the RA is contained within the vortex and highlighting the importance of organized atrial flow for efficient right heart function.^19,24,32^

Arvidsson *et al.*^23^ further describe the improved preservation of KE with rotational vs. non-rotational flow.^23^ They demonstrate a correlation between LA early diastolic KE and LV mass, suggesting the importance of the diastolic suction effect of the left ventricle in LV passive filling. This correlation was not true for the RV, indicating the importance of RA flow dynamics rather than ventricular forces in RV filling, preserving the kinetic energy of the blood pool and transferring this energy to the RV.^23^

### Strengths and limitations

The strength of this body of evidence lies in the consistent use of 4D flow CMR across multiple centres and diverse populations, in health and disease state, to explore right heart hemodynamics. There is consistency in the identification and alteration of RV flow components (decreased DF, increased RVo) in PH across different studies and subtypes (PAH, CTEPH, pre-PH). Similarly, age-related changes in RA flow and diastolic KE appear consistent. The correlation of 4D flow metrics with established functional parameters, invasive hemodynamics, and clinical outcomes strengthens their validity.

However, there are limitations to this review. Many studies had relatively small sample sizes, particularly for specific disease subgroups or age categories. Methodological heterogeneity in 4D flow acquisition and post-processing likely contributes to variability in reported absolute values, and there was significant heterogeneity across studies in which parameters were reported (*Table 3*). Most studies were cross-sectional, limiting causal inference or longer-term implications of flow parameters.

**Table 3:** Table detailing the CMR protocols used by the studies included within this systematic review

CMR Protocol					
No.	First author (year)	Vendor	Velocity Encoding	Spatial resolution (mm^3^)	Temporal resolution (ms)
1	Arvidsson (2013)	3T Achieva *(Philips Medical Systems, Best, the Netherlands)*	—	—	25–50
2	Arvidsson (2017)^5^	3T Achieva 1.5T Achieva *(Philips Medical Systems, Best, the Netherlands)*	100 cm/s	3.0 × 3.0 × 3.0	50
3	Barker (2020)^19^	1.5T Ingenia *(Philips Medical Systems, Best, the Netherlands)*	150 cm/s	3.0 × 3.0 × 3.0	40
4	Callaghan (2017)^8^	3T Skyra *(Siemens Healthcare, Erlangen, Germany)*	150–170 cm/s	2.5 × 2.5 × 2.5	—
5	Dewhurst (2020)^9^	3T Achieva *(Philips Medical Systems, Best, the Netherlands)*	150 cm/s	3.0 × 3.0 × 3.0	50–55
6	Fenster (2015)^24^	1.5T MAGNETOM Avanto *(Siemens Healthcare, Erlangen, Germany)*	100 cm/s	-	50
7	Fredriksson (2011)^10^	1.5T Achieva *(Philips Medical Systems, Best, the Netherlands)*	100 cm/s	3.0 × 3.0 × 3.0	49
8	Fredriksson (2016)^25^	3T Ingenia *(Philips Medical Systems, Best, the Netherlands)*	120 cm/s	2.8 × 2.8 × 2.8	—
9	Han (2015)^6^	1.5T Avanto *(Siemens Healthcare, Erlangen, Germany)*	125 cm/s	2.5 × 2.5 × 2.5	—
10	Nakaji (2021)^11^	3T MAGNETOM Skyra *(Siemens Healthcare, Erlangen, Germany)*	150 cm/s	1.8 × 1.8 × 4.0	68
11	Parikh (2017)^26^	3T Achieva *(Philips Medical Systems, Best, the Netherlands)*	150 cm/s	1.0 × 1.0 × 1.0	50–55
12	Wang (2021)^27^	1.5T MAGNETOM Aera *(Siemens Healthcare, Erlangen, Germany)*	100–150 cm/s	2.4 × 2.4 × 2.4	40
13	Wehrum (2018)^28^	3T MAGNETOM Trio *(Siemens Healthcare, Erlangen, Germany)*	150 cm/s	2.1 × 2.1 × 2.5	20
14	Xu (2022)^29^	1.5 MAGNETOM Aera *(Siemens Healthcare, Erlangen, Germany)*	100–150 cm/s	2.4 × 2.4 × 2.8	40
15	Xu (2023)^30^	1.5T MAGNETOM Aera *(Siemens Healthcare, Erlangen, Germany)*	100–150 cm/s	2.4 × 2.4 × 2.8	50
16	Zhao (2022)^31^	3T Ingenia *(Philips Medical Systems, Best, the Netherlands)*	150 (maximum 220) cm/s	3.0 × 3.0 × 3.0	—
		1.5T MAGNETOM Aera *(Siemens Healthcare, Erlangen, Germany)*	220 cm/s	3.0 × 3.0 × 3.0	—
17	Zhao (2023)^32^	3T Ingenia *(Philips Medical Systems, Best, the Netherlands)*	150 (maximum 220) cm/s	3.0 × 3.0 × 3.0	—
		1.5T MAGNETOM Aera *(Siemens Healthcare, Erlangen, Germany)*	220 cm/s	3.0 × 3.0 × 3.0	—

T, tesla.

### Future perspectives

The findings synthesized in this review suggest that 4D flow CMR offers valuable, qualitative and quantitative, clinically applicable markers of right heart hemodynamic function. Markers like RA flow dynamics, RV flow components and KE/EL show early promise for non-invasive assessment of right heart function and early identification of right heart dysfunction. Further work is required to standardize the acquisition and reporting of these parameters, while identifying normal values by age, gender, and ethnicity. Better understanding of the mechanisms and impact of right atrial flow dynamics would help explain its role in right heart dysfunction, with longitudinal data to investigate the long-term impact of these early markers of right heart disease. Further investigation into conditions directly impacting right atrial flow, including AF and tricuspid regurgitation, may present further insights into right atrial function and adaptation. An improved understanding of CS flow would also aid our understanding of RA function, especially in the current era of multiple implantable devices, yet to be approved, that interact with the CS. Lastly, it will be critical to demonstrate the incremental value provided by these measurements, above and beyond what standard 2 dimensional phase-contrast imaging and volumetric/functional CMR assessment provide, given the additional logistical considerations for both imaging acquisition and post-processing.

## Conclusion

4D flow CMR provides a powerful tool for non-invasive assessment of right heart hemodynamics. The quantification of flow patterns, components and energetics yields a comprehensive overview of right heart function that holds significant promise in improving the diagnosis, management, and prognostic stratification of right heart disease.

## Data Availability

All data analysed in this systematic review are derived from published studies and publicly available sources. No new data were generated or analysed for this review. Details of the search strategy, study selection, and data extraction are provided within the article and its supplementary materials.

## Lead author biography

Dr Alex Gall is a CMR Clinical Research Fellow with the 4D Lab at the University of East Anglia, UK. His research interests include using advanced 4D flow techniques to assess right heart physiology. He is also a Cardiology Registrar in the East of England.
